# Macroscopic types of intrahepatic cholangiocarcinoma and the eighth edition of AJCC/UICC TNM staging system

**DOI:** 10.18632/oncotarget.20932

**Published:** 2017-09-15

**Authors:** Ze-Wu Meng, Wei Pan, Hai-Jie Hong, Jiang-Zhi Chen, Yan-Ling Chen

**Affiliations:** ^1^ Department of Hepatobiliary Surgery, Fujian Medical University Union Hospital, Fuzhou, Fujian 350001, People's Republic of China; ^2^ Key Laboratory of Ministry of Education for Gastrointestinal Cancer, Fujian Medical University, Fuzhou, Fujian 350001, People's Republic of China

**Keywords:** intrahepatic cholangiocarcinoma, macroscopic type, TNM classification, clinicopathological characteristics, prognosis

## Abstract

The prognosis of patients with intrahepatic cholangiocarcinoma (ICC) is undefined among the different macroscopic types. This study evaluated the viability of the American Joint Committee on Cancer (AJCC) 8th edition staging classification for different macroscopic types. Utilizing the Surveillance, Epidemiology, and End Results (SEER) database, we enrolled a total of 2,679 eligible patients with an estimated 199 periductal infiltrating type of ICC (ICC-PI) patients and 2,480 mass-forming type of ICC (ICC-MF) patients. After conducting a multivariate Cox analysis, we found that the AJCC 8th edition staging system was suitable for ICC-MF patients but not for ICC-PI patients according to cancer-specific survival (CSS) and overall survival (OS). The main reason was the similar hazard ratio (HR) between the ICC-PI patients with stage I and stage II disease according to CSS (HR:0.969, *P* = 0.949) and OS (HR:0.832, *P* = 0.703). Moreover, we found that ICC-PI patients in AJCC stage I had a similar HR as ICC-MF patients in AJCC stage II according to CSS (HR: 1.208, *P* = 0.475) and OS (HR:1.206, *P* = 0.456). Therefore, we suggested that ICC-PI patients may be defined as T2, which is classified as stage II disease. This suggestion for the AJCC 8th edition staging system would be more suitable for different macroscopic types of ICC but requires further verification in prospective clinical trials.

## INTRODUCTION

The incidence and mortality of intrahepatic cholangiocarcinoma (ICC) has prominently increased over the past several decades both in the USA and worldwide [1]. As the second most common liver cancer, ICC is highly malignant and has an extremely poor prognosis [2–5]. The typical mass-forming (MF) type of ICC is a radial growth pattern that invades into the adjacent liver parenchyma, whereas the periductal infiltrating (PI) type of ICC demonstrates a diffuse and often ill-defined longitudinal growth pattern along the bile duct—an obvious clinicopathological difference with MF-type. The prognostic value of the growth pattern remains controversial, and the significance of this variable has not been compared with that of other prognostic factors [6, 7].

In the 6th edition staging system for hepatic malignancies established by the American Joint Committee on Cancer (AJCC)/Union for International Cancer Control (UICC), ICC staging is identical to that of hepatocellular carcinoma [8]. However, ICC has different carcinogenic mechanisms and biological behavior from hepatocellular carcinoma. Therefore, it was established as its own category in the revised staging system in the AJCC 7th edition staging manual [9], which is mainly derived from research conducted by Nathan et al. [10]. In the 7th edition staging system, the tumor growth patterns of the PI-type were first mentioned as the definition of primary tumor 4 (T4). After that, more research about the PI-type of ICC was reported. Uno M et al. [11] found that the percentage of intrahepatic metastases in ICC-PI patients was significantly lower than ICC-MF patients and that surgery could provide a more favorable outcome in ICC-PI patients. Imai K et al. [12] reported that patients with the PI-type of ICC without hilar invasion tend to have favorable surgical outcomes than patients with the MF-type. In contrast, Dover LL et al. [13] reported that patients with the MF-type of ICC exhibited significantly better survival than patients with the PI-type of ICC after surgical resection. Another study about the prognostic value of T4 (the tumor growth pattern of PI-type) was also reported. In 233 ICC patients who underwent curative resection, Takahiro Uenishi et al. [14] found that the survival curves failed to stratify the patients according to the 7th edition AJCC/UICC T classification because the survival prognoses of T2, T3, and T4 tumors were similar. Therefore, in the 8th edition staging system [7], the T4 category, which described the PI-type of ICC, was eliminated due to the controversial prognostic value of the growth pattern [6, 15–17]. The differences between the AJCC 6th, 7th and 8th edition staging manuals are described in Table 1.

**Table 1:** Different AJCC staging definitions for intrahepatic cholangiocarcinoma based on the AJCC 6th edition (2004), AJCC 7th edition (2010) and AJCC 8th edition (2017) staging systems

**AJCC staging classification (6th edition, 2004)**											**AJCC staging classification (7th edition, 2010)**					
T1			Single tumor without vascular invasion								T1	Solitary tumor without vascular invasion				
T2			Single tumor with vascular invasion or multiple tumors none more than 5 cm								T2a	Solitary tumor with vascular invasion				
											T2b	Multiple tumors, with or without vascular invasion				
T3			Multiple tumors more than 5 cm or tumors involving major branch of portal or hepatic veins								T3	Tumor perforating the visceral peritoneum or involving the local extra hepatic structures by direct invasion				
T4			Tumors with direct invasion of adjacent organs other than the gallbladder or with perforation of visceral peritoneum								T4	Tumor with periductal invasion				
N0			No regional lymph node metastasis								N0	No regional lymph node metastasis				
N1			Regional lymph node metastasis								N1	Regional lymph node metastasis present				
M0			No distant metastasis								M0	No distant metastasis				
M1			Distant metastasis								M1	Distant metastasis				
**AJCC staging classification (8th edition, 2017)**																
T1a							Solitary tumor ≤ 5 cm without vascular invasion									
T1b							Solitary tumor > 5 cm without vascular invasion									
T2							Solitary tumor with intrahepatic vascular invasion or multiple tumors, with or without vascular invasion									
T3							Tumor perforating the visceral peritoneum									
T4							Tumor involving the local extrahepatic structures by direct invasion									
N0							No regional lymph node metastasis									
N1							Regional lymph node metastasis present									
M0							No distant metastasis									
M1							Distant metastasis present									
**AJCC (6th edition, 2004)**					**AJCC (7th edition, 2010)**					**AJCC (8th edition, 2017)**					
**Stage**	**T**	**N**		**M**	**Stage**	**T**		**N**	**M**	**Stage**			**T**	**N**	**M**
I	T1	N0		M0	I	T1		N0	M0	I a			T1a	N0	M0
II	T2	N0		M0	II	T2a		N0	M0	I b			T1b	N0	M0
III a	T3	N0		M0		T2b		N0	M0	II			T2	N0	M0
III b	T4	N0		M0	III	T3		N0	M0	III a			T3	N0	M0
III c	Any T	N1		M0	IVa	T4		N0	M0	III b			T4	N0	M0
IV	Any T	Any N		M1		Any T		N1	M0				Any T	N1	M0
					IVb	Any T		Any N	M1	IV			Any T	Any N	M1

Abbreviations: AJCC, American Joint Committee on Cancer.

Because the mechanisms of carcinogenesis may differ among these macroscopic types [18], the MF-type of ICC often invades into the adjacent liver parenchyma, whereas the PI-type of ICC invades into the hepatic hilum. The growth and invasive patterns between these subtypes were all different. The present study was conducted to analyze differences in the clinicopathological factors and survival prognoses between the MF-type and PI-type of ICC using a large data set and to evaluate the viability of the AJCC 8th edition staging classification for the MF-type and PI-type. Finally, we proposed some modifications for the AJCC 8th edition staging system to render it more suitable for distinguishing the MF-type and PI-type of ICC.

## RESULTS

### Patient characteristics

In total, 2,679 patients from the SEER database with either pathologically or clinically confirmed ICC were included in this study (Table 2), including 199 cases of the ICC-PI subtype and 2,480 cases of the ICC-MF subtype. The median age at diagnosis was 67.0 years (range: 15–99 years), and the median CSS and OS were 10.1 and 9.4 months (range: 0–59 months for both), respectively. The comparison of clinicopathological characteristics of the ICC-PI and ICC-MF subtypes are summarized in Table 2. There were some differences in the characteristics between the two types, including race, AJCC stage (8th edition, 2017), and surgical status. Thus, we used the intergroup analysis to define the specific differences in the race and AJCC stage subgroups. The results indicated that higher percentage of black patients with ICC presented the PI-type the than MF-type (14.1% vs. 7.7%, respectively; *P* < 0.001). In addition, ICC-MF type patients represented a significantly higher percentage of AJCC stage II cases (21.5% vs. 14.1%, *P* = 0.013) and a lower percentage of AJCC stage IV cases (34.3% vs. 41.7%, *P* = 0.036). Moreover, ICC-PI type patients were more inclined to undergo surgery than ICC-MF type patients (33.7% vs. 26.3, *P* = 0.029). Other tumor characteristics, including age, sex, marital status, histologic grade, node stage, showed similar distributions between the two subtypes.

**Table 2:** Clinicopathological characteristics of patients with the MF-type and PI-type of intrahepatic cholangiocarcinoma

Characteristic	MF-type (*n* = 2480)	PI-type (*n* = 199)	Total (*n* = 2679)	P^a^
	No (%)	No (%)	No (%)	
Age (years)				0.878
≤ 60	753 (30.4)	62 (31.2)	815 (30.4)	
> 60	1727 (69.6)	137 (68.8)	1864 (69.6)	
Race				**0.005**
White	1942 (78.3)	149 (74.9)	2091 (78.1)	
Black	190 (7.7)	28 (14.1)	218 (8.1)	
Other^b^	348 (14.0)	22 (11.0)	370 (13.8)	
Sex				0.878
Male	1425 (57.5)	116 (58.3)	1541 (57.5)	
Female	1055 (42.5)	83 (41.7)	1138 (42.5)	
Marital status				0.350
Married	1442 (58.1)	123 (61.8)	1565 (58.4)	
Not married^c^	1038 (41.9)	76 (38.2)	1114 (41.6)	
Histologic grade				0.152
Grade I	123 (5.0)	4 (2.0)	127 (4.7)	
Grade II	517 (20.8)	52 (26.1)	569 (21.2)	
Grade III	463 (18.7)	38 (19.1)	501 (18.7)	
Grade IV	9 (0.4)	1 (0.5)	10 (0.5)	
Unknown	1368 (55.1)	104 (52.3)	1472 (54.9)	
AJCC stage (8th edition, 2017)				**0.043**
Stage I	597 (24.1)	48 (24.1)	645 (24.1)	
Stage II	533 (21.5)	28 (14.1)	561 (20.9)	
Stage III	498 (20.1)	40 (20.1)	538 (20.1)	
Stage IV	852 (34.3)	83 (41.7)	935 (34.9)	
Node stage				0.979
N0	1677 (67.6)	133 (66.8)	1810 (67.6)	
N1	706 (28.5)	57 (28.7)	763 (28.5)	
NX	97 (3.9)	9 (4.5)	106 (3.9)	
Surgery performed				**0.029**
Yes	652 (26.3)	67 (33.7)	719 (26.8)	
No	1828 (73.7)	132 (66.3)	1960 (73.2)	

Abbreviations: AJCC, American Joint Committee on Cancer. MF, mass-forming; PI, periductal infiltrating; NX: Regional lymph nodes could not be assessed.

^a^Bold type indicates statistical significance.

^b^Other includes Asian/Pacific Islander and American Indian/Alaskan native.

^c^Not married includes single, divorced, separated, unmarried or domestic partner, and widowed.

### Comparison of survival between the ICC-MF type and ICC-PI type

**Figure 1:**
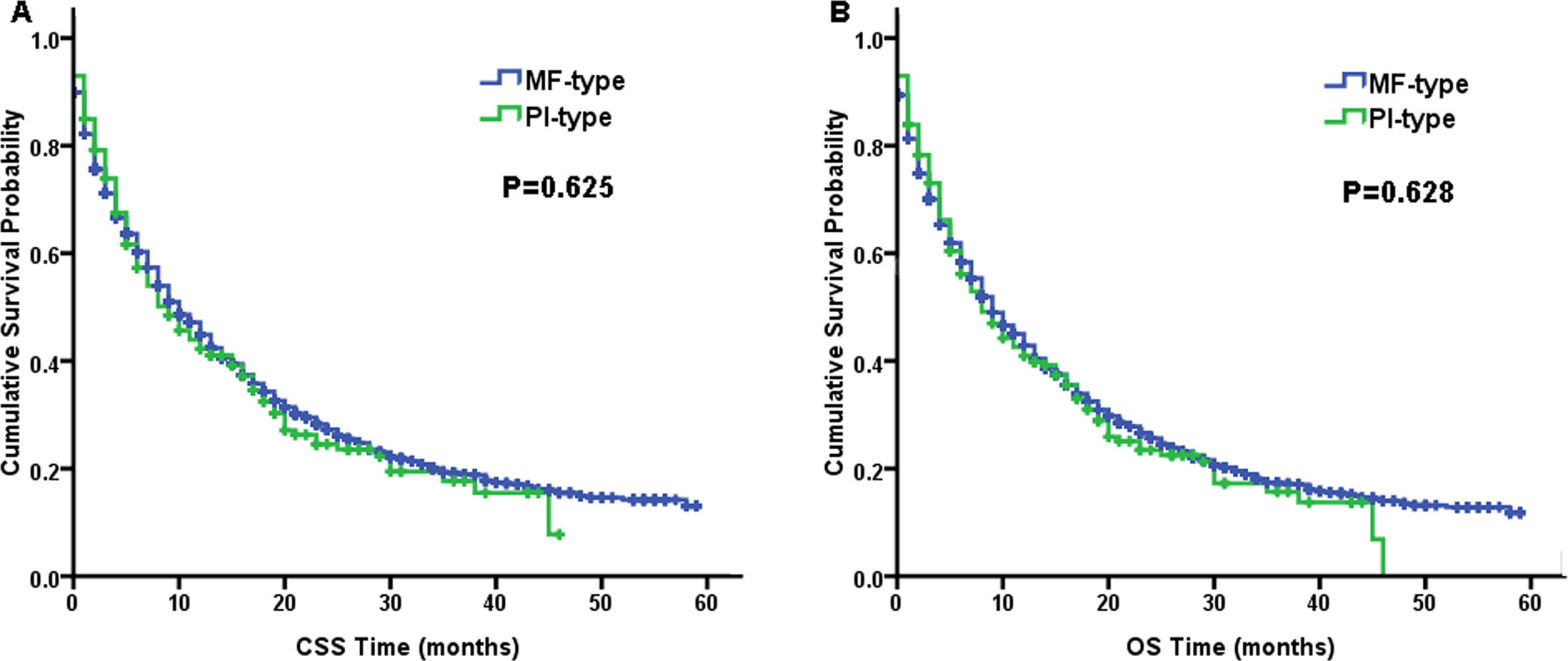
Kaplan–Meier curves of cancer-specific survival (CSS, **A**) and overall survival (OS, **B**) for the mass-forming type (MF-type) vs. periductal infiltrating type (PI-type) of intrahepatic cholangiocarcinoma.

There were no differences between the ICC-MF type and ICC-PI type regarding the survival prognosis. Figure 1 presents the Kaplan-Meier survival curves of CSS and OS for the two macroscopic types. The CSS (log-rank, *P* = 0.625) and OS (log-rank, *P* = 0.628) between these two ICC subtypes were similar.

**Figure 2:**
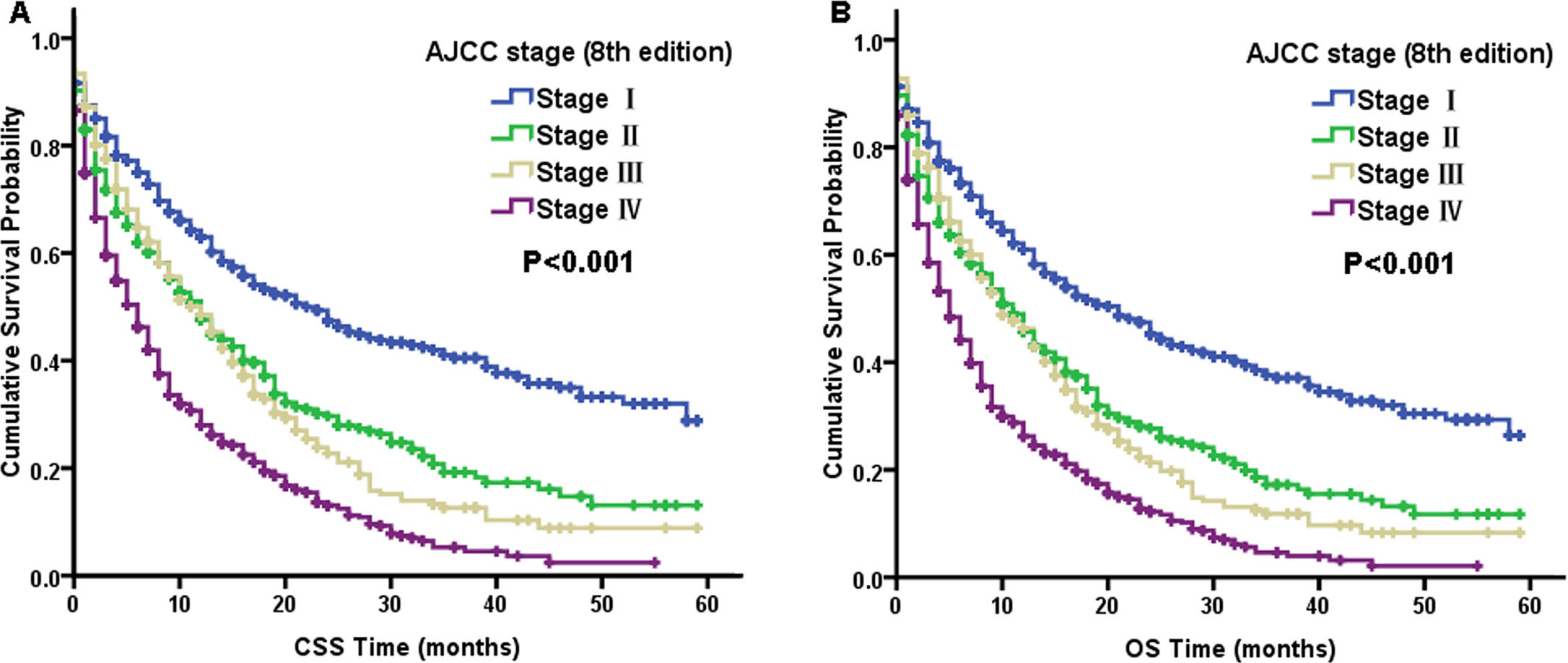
Kaplan–Meier curves of cancer-specific survival (CSS, **A**) and overall survival (OS, **B**) based on the AJCC stage of the mass-forming type (MF-type) of intrahepatic cholangiocarcinoma.

**Figure 3:**
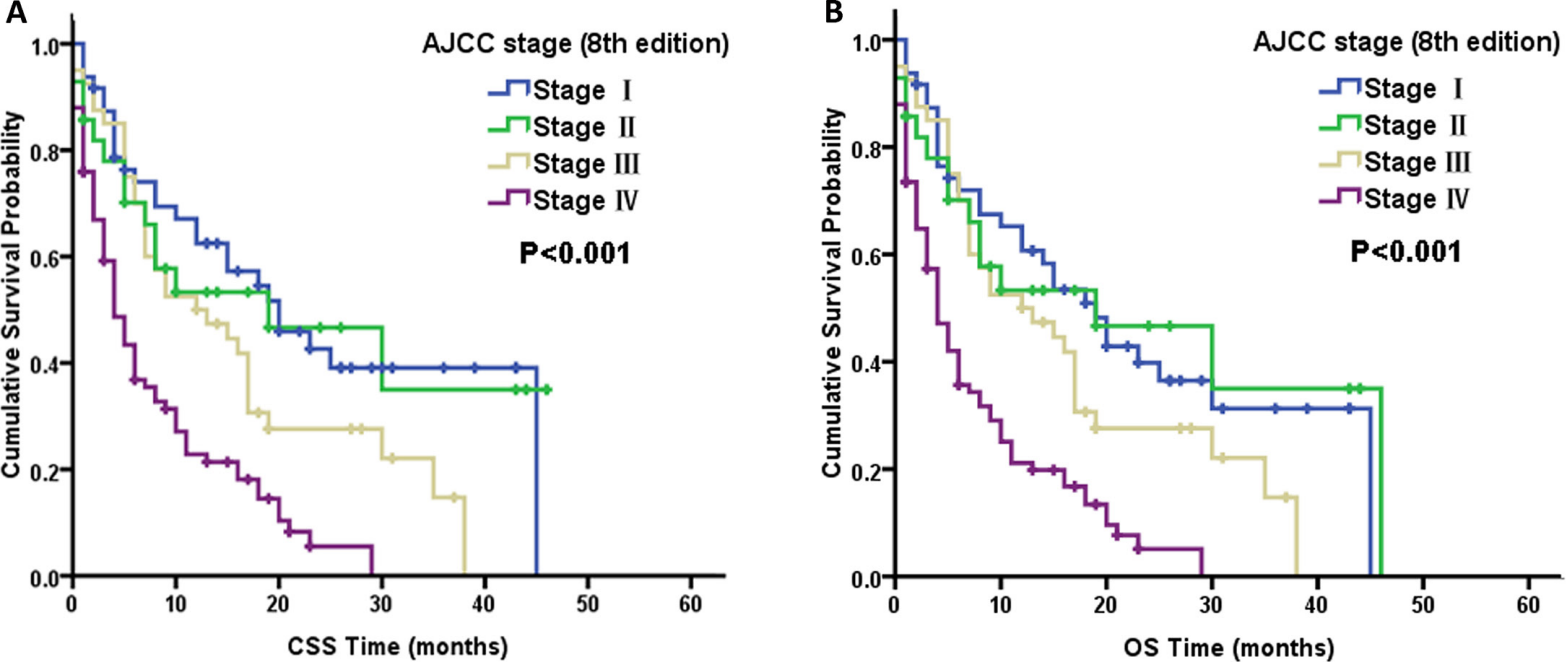
Kaplan–Meier curves of cancer-specific survival (CSS, **A**) and overall survival (OS, **B**) based on the AJCC stage of the periductal infiltrating type (PI-type) of intrahepatic cholangiocarcinoma.

Table 3 summarize the prognostic factors according to the CSS and OS results from multivariate Cox proportional hazard regression models for these two macroscopic types. As the AJCC staging system predominantly comprises node stages, we did not include node-stage variables in the multivariate Cox model because of their obvious correlation. Many factors, including older age, poor differentiation, and lack of surgical treatment, were all significantly associated with poor CSS and OS in the multivariate Cox analysis for these two macroscopic types (Table 3). After conducting subgroup analyses for the macroscopic subtypes using multivariate Cox models, we found that statistically significant differences existed among all the AJCC stages for ICC-MF patients according to CSS and OS. From Figure 2, the survival curves were also well differentiated by AJCC stage for ICC-MF patients. However, in ICC-PI type patients, the AJCC stage was no longer an independent prognostic factor for CSS and OS. It is also notable that the AJCC staging classification for ICC-PI patients overlapped between stage I and II disease according to CSS (log-rank, *P* = 0.762) and OS (log-rank, *P* = 0.972) (Figure 3).

**Table 3:** Multivariate Cox analysis of cancer-specific survival and overall survival for the different growth patterns of intrahepatic cholangiocarcinoma

Characteristic	Cancer-specific survival						Overall survival					
	MF-type			PI-type			PI-type					
	HR	95% CI	p^a^	HR	95% CI	p^a^	HR	95% CI	p^a^	HR	95% CI	p^a^
Age (years)												
≤ 60	1			1			1			1		
> 60	1.505	1.260–1.798	**< 0.001**	1.888	1.029–3.463	**0.040**	1.543	1.299–1.834	**< 0.001**	2.058	1.127–3.757	**0.019**
Race												
White	1			1			1			1		
Black	1.243	0.913–1.691	0.168	1.078	0.503–2.311	0.846	1.210	0.895–1.636	0.215	1.008	0.474–2.142	0.984
Other	0.942	0.742–1.197	0.626	1.235	0.546–2.790	0.612	0.989	0.789–1.240	0.925	1.364	0.627–2.969	0.434
Sex												
Male	1			1			1			1		
Female	0.887	0.748–1.053	0.171	0.804	0.451–1.432	0.458	0.880	0.746–1.038	0.130	0.710	0.404–1.247	0.233
Marital status												
Married	1			1			1			1		
Not married	1.093	0.922–1.296	0.303	0.838	0.467–1.504	0.554	1.112	0.944–1.310	0.202	0.842	0.477–1.485	0.552
Histologic grade												
Grade I + II	1			1			1			1		
Grade III + IV	1.574	1.333–1.859	**< 0.001**	2.583	1.483–4.499	**0.001**	1.560	1.329–1.832	**< 0.001**	2.398	1.400–4.107	**0.001**
AJCC stage (8th edition, 2017)												
Stage I	1			1			1			1		
Stage II	1.766	1.341–2.326	**< 0.001**	0.969	0.371–2.534	0.949	1.760	1.356–2.285	**< 0.001**	0.832	0.323–2.141	0.703
Stage III	2.176	1.657–2.858	**< 0.001**	1.837	0.847–3.987	0.124	2.073	1.597–2.691	**< 0.001**	1.543	0.729–3.267	0.257
Stage IV	2.397	1.846–3.113	**< 0.001**	1.971	0.864–4.496	0.107	2.315	1.804–2.971	**< 0.001**	2.018	0.909–4.478	0.084
Surgery performed												
Performed	1			1			1			1		
Not performed	4.115	3.321–5.098	**< 0.001**	2.897	1.424–5.891	**0.003**	3.938	3.210–4.830	**< 0.001**	2.784	1.392–5.569	**0.004**

Abbreviations: AJCC, American Joint Committee on Cancer; MF, mass-forming; PI, periductal infiltrating.

^a^ Bold type indicates statistical significance.

### Stratification analysis with AJCC stage subtype

We stratified the AJCC stage subtype to further validate the different outcomes affected by AJCC stage subtype between ICC-MF and ICC-PI cases. As shown in Table 4, the multivariate analysis revealed similar survival for AJCC stage II, III, and IV between ICC-MF and ICC-PI cases. However, in AJCC stage I cases, survival was poor in ICC-PI patients compared to that in ICC-MF patients according to CSS (with ICC-MF as the reference value: HR for ICC-PI, HR: 1.941, *P* = 0.014) and OS (HR: 1.985, *P* = 0.007). When we compared ICC-PI patients in AJCC stage I to ICC-MF patients in AJCC stage II, there was no difference in survival according to CSS (with ICC-MF in stage II as the reference value: HR for ICC-PI in stage I, HR: 1.208, *P* = 0.475) and OS (HR: 1.206, *P* = 0.456). These findings indicated that ICC-PI patients in stage I has a similar survival prognosis as ICC-PI and ICC-MF patients in stage II.

**Table 4:** Comparison of cancer-specific survival and overall survival between the MF-type and PI-type of intrahepatic cholangiocarcinoma after subgroup analyses using a multivariate Cox proportional hazard model

AJCC stage (8th edition, 2017)	Cancer-specific survival			Overall survival		
	HR	95% CI	p^a^	HR	95% CI	p^a^
stage I						
MF-type (*n* = 597)	1			1		
PI-type (*n* = 48)	1.941	1.141–3.302	**0.014**	1.985	1.202–3.280	**0.007**
stage II						
MF-type (*n* = 533)	1			1		
PI-type (*n* = 28)	1.188	0.515–2.739	0.687	1.038	0.452–2.383	0.931
stage III						
MF-type (*n* = 498)	1			1		
PI-type (*n* = 40)	1.296	0.728–2.307	0.378	1.214	0.685–2.153	0.506
stage IV						
MF-type (*n* = 852)	1			1		
PI-type (*n* = 83)	1.085	0.714–1.650	0.702	1.104	0.737–1.652	0.632
MF of stage II vs. PI of stage I						
stage II MF-type (*n* = 533)	1			1		
stage I PI-type (*n* = 48)	1.208	0.719–2.031	0.475	1.206	0.737–1.972	0.456

Abbreviations: AJCC, American Joint Committee on Cancer; MF, mass–forming; PI, periductal infiltrating.

^a^The *P* value was adjusted using a multivariate Cox proportional hazard regression model that accounted for age, race, sex, marital status, histologic grade, and surgery status. Bold type indicates statistical significance.

## DISCUSSION

The incidence of ICC is increasing, and the prognosis of ICC patients remains unfavorable. Since the recent release of the 8th edition of the AJCC staging system, there have been no reports on the advantage and applicability of this staging system. Using population-based data from a large cohort, we aimed to analyze the characteristics and outcomes of ICC-PI and ICC-MF patients and to evaluate the viability of the AJCC 8th edition staging classification for the MF and PI subtypes of ICC. The purpose of this study was to provide suggestions for staging ICC-MF and ICC-PI patients.

In total, 2,679 ICC patients were included in this study, 199 (7.4%) of which were had the PI subtype of ICC. This proportion was comparable to other studies [11, 15]. Table 2 shows that the PI-type of ICC is more prominent among black Americans, which is a new discovery. In addition, the percentage of the ICC-PI type cases at AJCC stage II was lower than that of the ICC-MF type cases. However, the proportion of ICC-PI type cases in AJCC stage IV was higher than that of ICC-MF type cases. This translates as fewer intrahepatic metastases but more distant metastases in the ICC-PI type. The characteristic of a high rate of distant metastasis for the ICC-PI type has not been reported to date. Moreover, ICC-PI type patients were more inclined to accept surgery than ICC-MF type patients—this is probably due to the lower incidence of intrahepatic metastases in the ICC-PI type [11].

Based on the results of this study, we found that the age, histologic grade, AJCC stage (8th edition), and surgical status were related prognostic factors (Table 3). This finding was previously reported in many other studies [19–21]. However, for the ICC-MF type and ICC-PI type, there were no significant associations with CSS and OS in the ICC patients. Figure 1 presents the survival curves of the two macroscopic types for CSS (log-rank, *P* = 0.625) and OS (log-rank, *P* = 0.628), both of which were similar. But in many other studies, the survival comparison results of the two macroscopic types were various. Some reported that the two macroscopic types of ICC have similar survival rates just like our study [6]. In addition, some reported that patients with the PI-type of ICC had significantly better survival than those with the MF-type of ICC [11, 12]. Moreover, there was also a report about the opposite result [13]. The different outcomes of these studies may be due to the characteristics of the ICC-PI type: fewer intrahepatic metastases but more distant metastases. ICC-PI type patients were more inclined to accept surgery than ICC-MF type patients, which was probably due to the lower incidence of intrahepatic metastases in the ICC-PI type [11]. Although we performed radical resection for localized ICC, the tumor may have distantly metastasized, which was not identified prior to surgery. This confounding factor leads to the unpredictable survival rates after surgery and nonsensical result above. For our study, the survival between the PI-type and the MF-type of ICC was similar in the entire ICC patient cohort.

After subgroup analysis for the macroscopic types using multivariate Cox models, we found that the AJCC 8th edition staging system was suitable for staging ICC-MF patients according to CSS and OS (Figure 2, Table 3). However, for ICC-PI patients, the AJCC staging system was no longer an independent prognostic factor for CSS and OS (Table 3). The main reason for this was the similar HR for CSS and OS between the stage I and stage II of ICC-PI patients. Figure 3 shows that the AJCC staging classification overlapped between stage I and II disease according to CSS and OS for ICC-PI patients. Therefore, we stratified the AJCC stage subtypes to further validate the different outcomes affected by AJCC stage subtype between ICC-MF and ICC-PI cases. As shown in Table 4, the multivariate analysis of AJCC stage I cases revealed that ICC-PI patients had poorer survival than ICC-MF patients according to CSS and OS. In addition, we found that the ICC-PI patient group in AJCC stage I disease had a similar HR as the ICC-MF patient group with AJCC stage II disease according to CSS and OS. These findings indicated that ICC-PI patients in stage I have a similar survival prognosis as ICC-PI and ICC-MF patients in stage II. The definition of stage I is a solitary tumor without vascular invasion. The main differences between stage I and stage II are vascular invasion and intrahepatic metastasis. Therefore, the staging system is suitable for ICC-MF patients due to its metastasizing characteristics of this subtype via the portal vein system [22]. However, the PI-type of ICC most commonly spreads via the lymphatic system [22]. Therefore, the definitions of AJCC stage I and stage II are not suitable for staging ICC-PI patients, which leads to similar survival prognoses between ICC-PI patients in stage I and ICC-PI and ICC-MF patients in stage II. Because ICC-PI patients in stage I have a similar survival prognosis as ICC disease exhibiting vascular invasion and intrahepatic metastasis (i.e., AJCC stage II) and the uncertainty of its own metastasis behavior, we suggest that the ICC-PI type be defined as a special type of intrahepatic metastasis and be categorized as stage T2 regardless of the presence or absence of intrahepatic metastasis. If a large-scale multicenter is conducted, it should be feasible to establish separate stages for ICC-PI due to its different growth and invasive behaviors.

This study is the first to evaluate the viability of the AJCC 8th edition staging classification for the MF-type and PI-type of ICC. In addition, we also propose a slight modification for staging the ICC-PI type in the AJCC 8th edition staging system. However, this study was limited by its retrospective nature, and the results need to be confirmed using additional large-scale studies.

## MATERIALS AND METHODS

### Patients

The Surveillance, Epidemiology, and End Results (SEER) [23] database (1973 to 2014) was used to identify ICC patients. Patients evaluated between 2010 and 2014 were chosen because both the 6th and 7th editions of the AJCC staging system were used to characterize the patients examined during this period. Since the release of the 8th edition of the AJCC staging system in 2017, it has not yet implemented in a large cohort of clinical patients. We used the data from the AJCC 6th and 7th editions staging classifications to transform to the AJCC 8th edition staging classification to correlate the stages. The corresponding conversion method is described in Table 5. Patients were identified based on the International Classification of Diseases for Oncology (3rd editions) [24]. The following coding was used: the primary site code for the liver (22.0); the histology code for cholangiocarcinoma (8160); the primary site code for the intrahepatic bile duct (22.1); the histology codes for malignant neoplasm (8000), malignant tumor cells (8001), carcinoma (8010), undifferentiated carcinoma (8020), adenocarcinoma (8140), and cholangiocarcinoma (8160); and a behavior code (3-malignant tumor). Finally, 2,679 cases were included in our study. The SEER 8.3.4 registry research database was utilized to generate a listing of ICC cases, and the following variables were extracted: site recode (intrahepatic bile duct), behavior recode for analysis (malignant), age, race, sex, marital status at diagnosis, growth patterns (i.e., MF-type, PI-type), histological grade, AJCC stage (6th edition, 2004), AJCC stage (7th edition, 2010), node stage, surgical status (yes, no), SEER cause-specific death classification, vital status recode, and survival (months). Age at diagnosis was stratified as ≤ 60 years and > 60 years. Race was recoded as white, black, or other (includes Asian/Pacific Islander and American Indian/Alaskan native). Marital status was categorized as married or not married (i.e., single, divorced, separated, unmarried or domestic partner, widowed). Tumor growth patterns were classified as MF-type and PI-type. Histological grades were classified as grade I (well differentiated), grade II (moderately differentiated), grade III (poorly differentiated), grade IV (undifferentiated), and unknown. The cancer-specific survival (CSS) was defined as the time from the date of diagnosis to the date of either death due to ICC, and overall survival (OS) was calculated from the date of diagnosis to the date of death due to any cause. Patients who were alive were censored on the date of last contact for both outcomes. The characteristics of the 2,679 patients with ICC between the different growth patterns are described in Table 2.

**Table 5:** The corresponding stage definitions for intrahepatic cholangiocarcinoma from the AJCC 6th edition (2004) and AJCC 7th edition (2010) and AJCC 8th edition (2017)

AJCC staging classification		
8th edition (2017)	7th edition (2010)	6th edition (2004)
I	I	I
II	II	II+ III a
III	III + IV a–T4N0M0	III b + III c
IV	IV b	IV

Abbreviations: AJCC, American Joint Committee on Cancer.

### Statistical analysis

All computations were performed using SPSS version 13.0 for Windows (SPSS Inc, IL, USA). The chi-square test and one-way analysis of variance were employed to compare the demographic and clinical characteristics of the ICC-MF and ICC-PI groups. CSS and OS were analyzed using Kaplan-Meier curves, and log-rank tests were used to evaluate the correlation between the AJCC 8th edition staging system and growth patterns. Multivariate analyses for each staging system and different growth patterns were completed using Cox proportional hazards regression models controlling for growth patterns, age, race, sex, marital status at diagnosis, histological grade, and surgical status. The hazard ratio (HR) and 95% confidence interval (CI) were calculated. All *P* values were two-sided, and values less than 0.05 were considered statistically significant.

### Ethics statement

For access to the SEER database, informed consent was not required, but a Data-Use Agreement for the SEER 1973–2014 Research Data File was completed.

## Abbreviations

ICC: intrahepatic cholangiocarcinoma
MF: mass-forming
PI: periductal infiltrating
AJCC: American Joint Committee on Cancer
UICC: Union for International Cancer Control
TNM: tumor–node–metastasis
CSS: cancer-specific survival
OS: overall survival
SEER: Surveillance, Epidemiology, and End Results
HR: hazard ratio
CI: confidence interval.
